# Higher serum albumin-corrected calcium levels are associated with revascularization and poor outcome after mechanical thrombectomy

**DOI:** 10.1186/s12883-022-02856-2

**Published:** 2022-09-02

**Authors:** Xinwei He, Baomei Lin, Taotao Tao, Qiuyue Chen, Jinhua Wang, Jiaolei Jin

**Affiliations:** 1 Department of Neurology, Taizhou Central Hospital (Taizhou University Hospital), 999 Donghai Road, Taizhou, 318000 China; 2 Department of Neurology, Huanggang Central Hospital, Huanggang, 438000 Hubei China

**Keywords:** Stroke, Mechanical thrombectomy, Outcome, Revascularization, Serum calcium

## Abstract

**Background:**

Serum calcium abnormalities have been determined to be associated with the risk and outcome of stroke. The aim of the present study was to examine the associations of serum calcium with vascular recanalization, symptomatic intracranial haemorrhage and functional outcome in stroke patients after mechanical thrombectomy.

**Methods:**

A total of 192 patients treated with mechanical thrombectomy for anterior circulation large vessel occlusion were consecutively included from August 2017 to June 2021. Serum calcium levels were measured on admission, and albumin-corrected calcium levels were calculated for subsequent analysis. Successful arterial revascularization was defined as a modified Thrombolysis in Cerebral Infarction scale score ≥ 2b. Symptomatic intracranial haemorrhage was assessed according to the European Cooperative Acute Stroke Study (ECASS) III criteria. Poor functional outcome was defined as a modified Rankin Scale score > 2 at 3 months.

**Results:**

Patients with poor outcomes had higher albumin-corrected calcium levels than patients with good outcomes before (2.20 (2.10, 2.30) mmol/L vs. 2.13 (2.04, 2.24) mmol/L, *P* = 0.002), and after adjusting for other factors (AOR 95% CI, 1.812 (1.253, 2.621), *P* = 0.002). Patients with unsuccessful recanalization had higher albumin-corrected calcium levels than those with recanalization (2.26 (2.09, 2.46) mmol/L vs. 2.17 (2.07, 2.27) mmol/L, *P* = 0.029), and after adjusting for other factors (AOR 95% CI, 2.068 (1.214, 3.524)), *P* = 0.008). No association was found between albumin-corrected calcium and symptomatic intracranial haemorrhage.

**Conclusions:**

Higher serum albumin-corrected calcium levels are independently associated with revascularization and poor outcome in stroke patients after mechanical thrombectomy.

**Supplementary Information:**

The online version contains supplementary material available at 10.1186/s12883-022-02856-2.

## Introduction

Stroke is one of the major causes of disability and death in adults worldwide [1, 2]. Ischaemic stroke caused by arterial occlusion is responsible for the majority of stroke cases [1, 2]. Mechanical thrombectomy is recommended as the treatment of choice for patients with stroke due to large vessel occlusion, which has markedly changed hyperacute stroke management [1–3].

Calcium is one of the most abundant minerals in the body and is essential to multiple body functions, including bone health, muscle contraction, nervous system function and the coagulation pathway [4, 5]. Serum calcium levels are maintained within a narrow range through regulation of the calcium homeostasis system [4–6]. Approximately half of the calcium in serum is in its active ionized form, and the rest is bound to albumin or other complexes [4–6].

Previous studies have confirmed the relationship between serum calcium and the progressive risk and outcome of cerebral vascular disease [6, 7]. Lower serum calcium levels are associated with a higher increased risk of haematoma expansion and poor outcome after intracranial haemorrhage [8, 9]. In addition, lower serum calcium levels at diagnosis are significantly associated with ruptured aneurysms [10]. The association between serum calcium and the outcome of ischaemic stroke is still controversial in different populations [11–13]. Moreover, the relationships between serum calcium levels and the clinical characteristics and outcomes of stroke patients after mechanical thrombectomy remain unknown.

Thus, the aim of the present study was to examine the associations of albumin-corrected calcium levels with vascular recanalization, symptomatic intracranial haemorrhage (sICH) and functional outcome in stroke patients after mechanical thrombectomy.

## Methods

### Study population

**Fig. 1:**
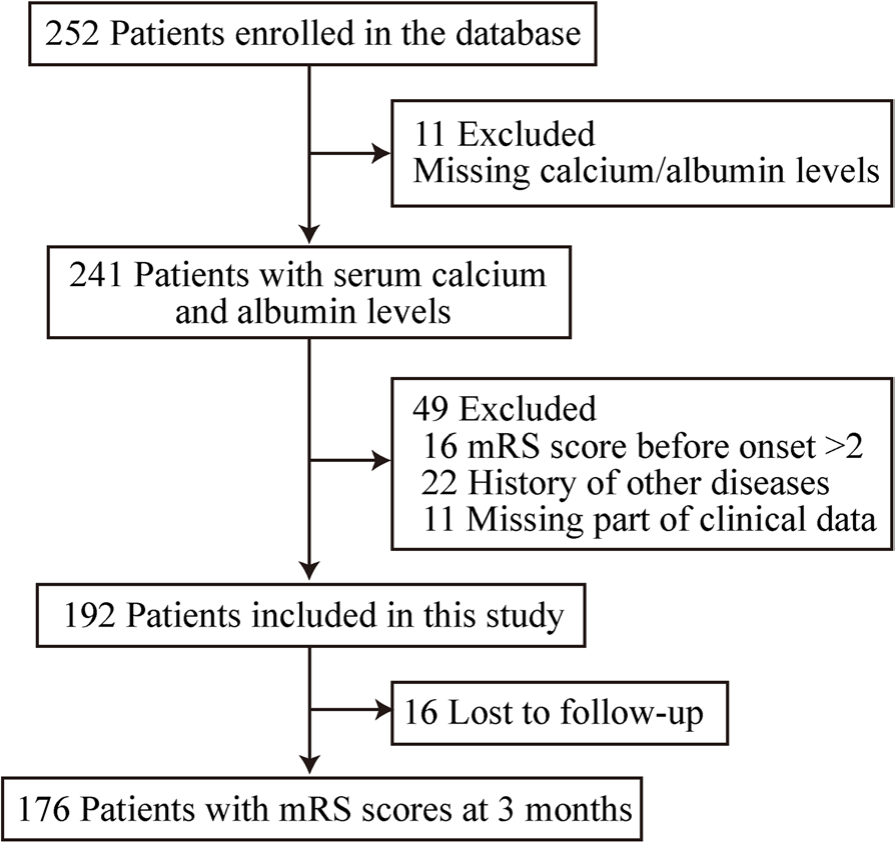
Study flowchart

We performed a retrospective analysis of a mechanical thrombectomy database at our hospitals from August 2017 to June 2021. The inclusion criteria were as follows: (1) patients with anterior circulation large vessel occlusion who underwent mechanical thrombectomy, and (2) serum calcium levels measured on admission. A total of 241 patients were consecutively included (Supplementary Table 1). The exclusion criteria were as follows: (1) modified Rankin Scale (mRS) score before onset > 2 (*n* = 16); (2) history of acute or chronic infectious diseases, severe liver or kidney insufficiency, and malignant tumours (*n* = 22); and (3) most clinical data were missing (*n* = 11). In total, 192 patients were enrolled in this study. The study flowchart is shown in Fig. 1.

The retrospective study was in accordance with the ethical guidelines of the 1975 Declaration of Helsinki. Written informed consent for participation was waived by the Medical Ethics Committee of Taizhou Central Hospital (approval number, 2021 L-09-12) and Huanggang Central Hospital (approval number, HGYY-KY-2021-008), because of the retrospective study based on routine clinical data and cannot identify the patient’s information.

### Baseline data and assessment

For all patients, we recorded demographic information and stroke risk factors (including hypertension, diabetes mellitus, dyslipidaemia, atrial fibrillation, previous stroke, smoking and alcohol consumption). Additionally, clinical features on admission and treatment procedures (including site of occlusion, intravenous thrombolysis, the National Institutes of Health Stroke Scale (NIHSS) score [14], the Alberta Stroke Program Early CT Score (ASPECTS) [15], number of passes, time from onset to revascularization, and periprocedural complications) were collected.

Vascular risk factors were identified as follows, hypertension (systolic blood pressure ≥ 140 mmHg, or diastolic blood pressure ≥ 90 mmHg at discharge or use of antihypertensive med use), diabetes mellitus (fasting blood glucose > 7.0 mmol/L, or hemoglobin A1c > 6.5%, or self-reported diabetes mellitus, or diabetes med use), hyperlipidemia (serum triglycerides > 1.7 mmol/L, low-density lipoprotein > 3.4 mmol/L, high-density lipoprotein cholesterol < 0.8 mmol/L, or antihyperlipidemic med use).

Blood samples were collected on admission and serum calcium levels were determined. In order to estimate free ionized calcium levels, albumin-corrected calcium was calculated by a modified formula, as follows: total serum calcium (mmol/L) + [40 -albumin (g/dL)] × 0.018 [16].

### Characteristics and outcomes of stroke

The stroke subtype was defined using the Trial of Org 10,172 in Acute Stroke Treatment (TOAST) criteria [17]. The modified Thrombolysis in Cerebral Infarction (mTICI) score was used to assess revascularization after mechanical thrombectomy and a mTICI score ≥ 2b was considered indicative of complete revascularization [15]. sICH was assessed according to the European Cooperative Acute Stroke Study (ECASS) III criteria (any intracranial hemorrhage associated with clinical deterioration, as defined by an increase of ≥4 points on the NIHSS, or that led to death) [18]. The clinical outcome measure was functional outcome assessed with the mRS score at 3 months after stroke by a specialized research nurse, and a poor outcome was defined as an mRS score > 2 [19].

### Statistical analysis

Categorical variables are expressed as frequencies (percentages), and continuous variables are expressed as the means ± standard deviations or medians (interquartile range [IQR]) values, as appropriate. Differences between the groups were analysed using the chi-square test or Fisher’s exact test for categorical variables and a *t*-test or the Mann-Whitney *U* test for continuous variables, as appropriate. Multivariate analyses were performed using binary logistic regression models, adjusted for potential influencing factors in the univariate analyses (*P* < 0.1). Albumin-corrected calcium was equally divided into quartiles in the logistic regression. All data were analysed using SPSS 20.0 (IBM, Chicago, IL, USA). A two-sided *P* < 0.05 was considered indicative of statistical significance.

## Results

### Baseline characteristics

In total, 192 consecutive patients with acute stroke of the anterior circulation treated with mechanical thrombectomy were included. The baseline characteristics of the study population are shown in Table 1. The mean age was 71.8 ± 10.5 years, 132 (68.7%) of the patients were men, the NIHSS score at baseline was 14 (10, 18), the ASPECT score at baseline was 8 (7, 9). 123 (64.1%) patients received intravenous thrombolysis treatment and the onset to puncture time was 335 (255, 410) minutes.

**Table 1:** Basic characteristics of study patients

Variables	*n* = 192
Age, years	71.8 ± 10.5
Male, n (%)	132 (68.7%)
Risk factors, n (%)	
Hypertension	128 (66.7%)
Diabetes mellitus	57 (29.7%)
Dyslipidemia	121 (63.0%)
Ischemic cardiopathy	58 (30.2%)
Arrhythmia	78 (40.6%)
Stroke history	41 (21.4%)
Hypertension med use	74 (38.5%)
Diabetes med use	25 (13.0%)
Smoking	80 (41.7%)
Drinking	32 (16.7%)
Laboratory results	
Fasting glucose, mmol/L	6.9 (5.5, 9.2)
Triglycerides, mmol/L	1.30 (0.90, 1.72)
Total cholesterol, mmol/L	4.29 (3.61, 5.20)
Homocysteine, mmol/L	13.0 (10.3, 18.0)
C-reactive protein, mg/L	8.0 (3.7, 18.6)
Albumin, g/L	37.5 (34.8, 40.5)
Calcium, mmol/L	2.11 (2.01, 2.23)
Stroke evaluation	
NIHSS at baseline	14 (10, 18)
ASPECT at baseline	8 (7, 9)
Site of vessel occlusion，n(%)	
ICA	74 (38.5%)
M1-MCA	104 (54.2%)
Others	14 (7.3%)
Procedure	
Onset to puncture time, min	335 (255, 410)
Onset to recanalization, min	385 (320, 450)
Successful recanalization, n (%)	172 (89.6%)
Intravenous thrombolysis, n (%)	123 (64.1%)
Number of passes	2 (1, 2)
sICH, n(%)	23 (12.0%)
TOAST subtype, n (%)	
Large-artery atherosclerosis	86 (44.8%)
Cardioembolism	87 (45.3%)
Other determined/undetermined	19 (9.9%)

*Abbreviation*: *ICA* Internal carotid artery, *M1-MCA* M1 of middle cerebral artery, *NIHSS* National Institutes of Health Stroke Scale, *ASPECT* Alberta Stroke Program Early CT Score, *sICH* Symptomatic intracranial hemorrhage, *TOAST* Trial of Org 10,172 in Acute Stroke Treatment

Calcium level was weakly correlated with fasting glucose (*R* = -0.166, *P* = 0.023, Table 2) and NIHSS at baseline (*R* = 0.160, *P* = 0.026, Table 2). Albumin-corrected calcium level was weakly correlated with fasting glucose (*R* = -0.166, *P* = 0.023, Table 2) and NIHSS at baseline (*R* = 0.160, *P* = 0.026, Table 2). Participants with higher concentrations of albumin-corrected calcium were more likely to have ICA occlusion (Supplementary Table 2).

**Table 2:** Correlation between calcium level and baseline characteristics of the patients

Variables	Calcium level		Albumin-corrected calcium level	
	R	*P*	R	*P*
Age, years	0.290	0.077	0.081	0.267
Fasting glucose, mmol/L	-0.166	**0.023**	−0.081	0.266
Triglycerides, mmol/L	0.128	0.079	0.006	0.932
Total cholesterol, mmol/L	0.088	0.229	0.061	0.404
Homocysteine, mmol/L	0.131	0.086	0.167	**0.029**
C-reactive protein, mg/L	−0.092	0.215	0.051	0.494
NIHSS at baseline	0.160	**0.026**	0.157	**0.030**
ASPECT at baseline	−0.138	0.057	−0.084	0.246

*Abbreviation*: *NIHSS* National Institutes of Health Stroke Scale, *ASPECT* Alberta Stroke Program Early CT Score

### Albumin-corrected calcium and arterial revascularization

**Fig. 2:**
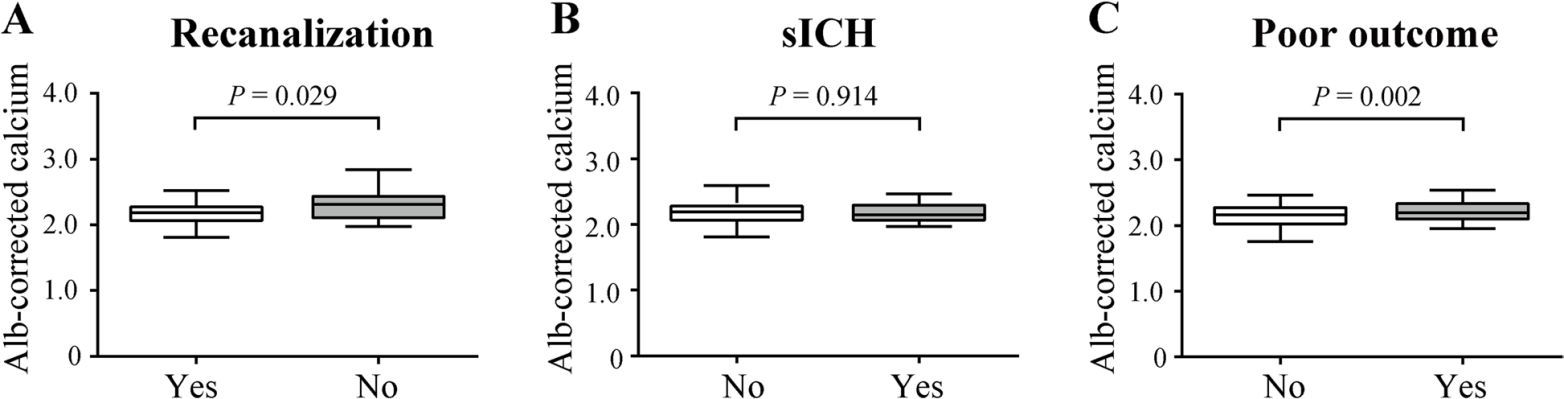
Association of albumin-corrected calcium and revascularization, sICH, functional outcome. **A** Compare between patients with or without recanalization. **B** Compare between patients with or without sICH. **C** Compare between patients with or without poor outcome. Abbreviation, Alb, Albumin, sICH, symptomatic intracranial hemorrhage

Complete arterial revascularization was achieved in 172 (89.6%) patients. Patients with unsuccessful recanalization had higher albumin-corrected calcium than patients with revascularization (2.26 (2.09, 2.46) mmol/L vs. 2.17 (2.07, 2.27) mmol/L, *P* = 0.029, Table 3, Fig. 2A). After adjusting for diabetes mellitus, arrhythmia, stroke history, smoking, homocysteine, number of passes, ICA occlusion, and TOAST subtype; Supplementary Table 3), the difference was still statistically significant (AOR 95% CI, 2.068 (1.214, 3.524)), *P* = 0.008).

**Table 3:** Association of albumin-corrected calcium and revascularization, sICH, and functional outcome

	Albumin-corrected calcium, mmol/L		*P* value	Adjusted OR 95%CI	*P* value
	Yes	No			
Recanalization	2.26 (2.09, 2.46)	2.17 (2.07, 2.27)	0.029	0.484 (0.284, 0.824) ^a^	0.008
sICH	2.15 (2.05, 2.26)	2.18 (2.08, 2.30)	0.914	/	/
Poor outcome	2.20 (2.10, 2.30)	2.13 (2.04, 2.24)	0.002	1.812 (1.253, 2.621) ^b^	0.002

Albumin-corrected calcium was equally divided into quartiles in the logistic regression

^a^After adjusting for diabetes mellitus, arrhythmia, stroke history, smoking, homocysteine, number of passes, ICA occlusion, and TOAST subtype

^b^Adjust adjusting for age, hypertension, diabetes mellitus, ischemic cardiopathy, arrhythmia, smoking, NIHSS scores, number of passes, homocysteine, and sICH

*Abbreviation*: *ICA* Internal carotid artery, *sICH* Symptomatic intracranial hemorrhage, *OR* Odds ratio, *95%CI* 95% confidence interval

### Association of albumin-corrected calcium with sICH and functional outcome

Of the 192 patients, 176 (91.7%) were followed up at 3 months, and 110 (62.5%) of them showed poor outcomes. Basically, patients with poor outcomes have higher albumin-corrected calcium levels than those with good outcomes (2.20 (2.10, 2.30) mmol/L vs. 2.13 (2.04, 2.24) mmol/L, *P* = 0.002, Table 3, Fig. 2C). After adjusting for confounders between groups (including age, hypertension, diabetes mellitus, ischemic cardiopathy, arrhythmia, smoking, NIHSS and ASPECT scores, ICA occlusion, number of passes, homocysteine, and sICH; Supplementary Table 4), the difference was still statistically significant (AOR 95% CI, 1.765 (1.239, 2.514), *P* = 0.002).

sICH was observed in 23 (12.0%) patients. No difference was found between patients with or without sICH (2.15 (2.05, 2.26) mmol/L vs. 2.18 (2.08, 2.30) mmol/L, *P* = 0.914, Table 3, Fig. 2B).

## Discussion

This is the first study to investigate the relationships between albumin-corrected calcium levels and clinical characteristics and functional outcome in stroke patients after mechanical thrombectomy. The major new findings were as follows: 1) patients in whom with unsuccessful recanalization had higher albumin-corrected calcium levels than those with complete arterial revascularization; 2) no relationship was found between albumin-corrected calcium and sICH; and 3) patients with poor outcomes have higher albumin-corrected calcium levels than those with good outcomes.

We found that higher albumin-corrected calcium levels were associated with unsuccessful arterial recanalization. High calcium levels are linked to accelerated vascular atherosclerosis and calcification, which is often associated with increased vascular stiffness and reduced vascular compliance [20, 21]. The distribution, pattern and degree of intracranial carotid artery atherosclerosis and calcification may affect the advancement of catheters and stentrievers, thereby reducing the chances of successful embolectomy, which in turn has a negative impact on recanalization.

We found that albumin-corrected calcium had no association with sICH. Some previous studies have discussed the association between calcium and ICH after stroke. A study found that lower admission serum total calcium levels were independently related to haemorrhagic transformation after intravenous thrombolysis [22]. The authors considered mediation of the extracellular coagulation cascade by ionized calcium via the activation of several coagulation factors, which contributes to the conversion of prothrombin to thrombin, to be a possible mechanism [22]. Since albumin levels have a major effect on total calcium levels without affecting physiologically important ionized calcium, it is better to correct calcium to albumin levels [4–6]. Furthermore, some studies have demonstrated that albumin-corrected calcium is a better parameter for evaluating the effect of calcium at the cellular level than serum calcium [12, 23]. Another study found that albumin-corrected calcium had no association with haemorrhagic transformation in patients without intravenous thrombolysis [24], which is similar to our study. Notably, 64.1% of patients in our study received intravenous thrombolysis. Another potential explanation for this finding is the relatively small sample size; there were 23 (12.0%) patients with sICH, which prevented us from detecting potential associations.

There is a growing concern about the impact of serum calcium levels on prognostic significance in terms of neurologic outcome and mortality after acute ischaemic stroke. However, the conclusion is still controversial. Recent studies found that higher albumin-corrected calcium levels are related to a poorer short-term outcome and an increased risk of long-term mortality after ischaemic stroke [11, 12]. In contrast, earlier studies found that elevated calcium levels obtained between 72 and 96 hours after ischaemic stroke are a good prognostic factor for better 3-month functional outcome, while early calcium levels did not have predictive value [13]. Interestingly, another study found that increasing serum calcium levels at both extremes are a marker of mortality in acute stroke patients [12].

We found that higher albumin-corrected calcium levels were associated with poor functional outcome in stroke patients after mechanical thrombectomy. The underlying biological mechanism has not been established, and several explanations are possible. First, as mentioned above, high calcium levels lead to vascular calcification and atherosclerosis, indicating a worse cerebrovascular basis. Second, experimental studies have demonstrated that calcium ions are ubiquitous intracellular messengers during and immediately after an ischaemic period, involving in related pathophysiological processes [25, 26]. Further studies are needed to assess whether and how circulating calcium is involved in pathophysiological processes in stroke patients after mechanical thrombectomy.

Some limitations of our study should be acknowledged. First, our study was a retrospective analysis with small sample. Multicentre prospective studies with direct measurement of calcium levels at different times after stroke are better. Second, we cannot obtain calcium levels before stroke, and due to the acute nature of the disease, some patients received treatment before referral, which may affect calcium levels. Third, some unmeasured or residual confounding factors may not be captured in this study, such as calcium agent use, information regarding stroke location and volume and postdischarge medical care quality. Finally, we did not completely measure the distribution, pattern and degree of intracranial atherosclerotic changes, and the potential mediation effect of atherosclerosis on the link between calcium levels and clinical outcome was not specifically addressed.

## Conclusions

In conclusion, our findings corroborated and extended previous evidence of a relationship between serum calcium and stroke. Our preliminary data suggested that serum albumin-corrected calcium on admission is a potential prognostic biomarker in stroke patients after mechanical thrombectomy.

## Supplementary Information

**Additional file 1: Supplementary Table 1.** Basic characteristics of study patients. **Supplementary Table 2.** Baseline characteristics of the patients according to quartiles of albumin corrected calcium level. **Supplementary Table 3.** Characteristics of patients with or without arterial revascularization. **Supplementary Table 4.** Characteristics of patients with good or poor outcome.

## Data Availability

The datasets used and/or analyzed during the current study do not contain identifiable data and are available from the corresponding author on reasonable request.

## Abbreviations

sICH: Symptomatic intracranial haemorrhage
mRS: Modified Rankin Scale
NIHSS: National Institutes of Health Stroke Scale
ASPECTS: Alberta Stroke Program Early CT Score
TOAST: Trial of Org 10,172 in Acute Stroke Treatment
mTICI: mThrombolysis in Cerebral Infarction
ECASS: European Cooperative Acute Stroke Study
