# Supplementary material for: Higher serum albumin-corrected calcium levels are associated with revascularization and poor outcome after mechanical thrombectomy
Source: BMC Neurol. 2022 Sep 2;22:330. doi: 10.1186/s12883-022-02856-2 (PMC9438214; doi:10.1186/s12883-022-02856-2)
Supplement: Supplementary file 1 — Additional file 1: Supplementary Table 1. Basic characteristics of study patients. Supplementary Table 2. Baseline characteristics of the patients according to quartiles of albumin corrected calcium level. Supplementary Table 3. Characteristics of patients with or without arterial revascularization. Supplementary Table 4. Characteristics of patients with good or poor outcome. [file 12883_2022_2856_MOESM1_ESM.docx]

**Supplemental materials**

**Supplementary Table 1. Basic characteristics of study patients**

| Variables | | All patients  (n=241) | Study patients  (n=192) | *P* |
| --- | --- | --- | --- | --- |
| Age, years | | 71.8 ± 10.5 | 71.8 ± 10.5 |  |
| Male, n (%) | | 160 (66.4%) | 132 (68.7%) | 0.603 |
| Risk factors, n (%) | |  |  |  |
|  | Hypertension | 164 (68.0%) | 128 (66.7%) | 0.760 |
|  | Diabetes mellitus | 84 (34.9%) | 57 (29.7%) | 0.254 |
|  | Dyslipidemia | 149 (61.8%) | 121 (63.0%) | 0.799 |
|  | Ischemic cardiopathy | 74 (30.7%) | 58 (30.2%) | 0.911 |
|  | Arrhythmia | 96 (39.8%) | 78 (40.6%) | 0.868 |
|  | Stroke history | 57 (23.7%) | 41 (21.4%) | 0.570 |
|  | Hypertension med use | 99 (41.1%) | 74 (38.5%) | 0.592 |
|  | Diabetes med use | 35 (14.5%) | 25 (13.0%) | 0.653 |
|  | Smoking | 110 (45.6%) | 80 (41.7%) | 0.407 |
|  | Drinking | 38 (15.8%) | 32 (16.7%) | 0.801 |
| Laboratory results | |  |  |  |
|  | Fasting glucose, mmol/L | 6.6 (5.4, 8.7) | 6.9 (5.5, 9.2) | 0.968 |
|  | Triglycerides, mmol/L | 1.30 (0.94, 1.81) | 1.30 (0.90, 1.72) | 0.981 |
|  | Total cholesterol, mmol/L | 4.29 (3.63, 5.15) | 4.29 (3.61, 5.20) | 0.791 |
|  | Homocysteine, mmol/L | 13.0 (10.3, 17.5) | 13.0 (10.3, 18.0) | 0.982 |
|  | C-reactive protein, mg/L | 6.7 (3.7, 20.3) | 8.0 (3.7, 18.6) | 0.550 |
|  | Albumin, g/L | 38.1 (35.4, 41.2) | 37.5 (34.8, 40.5) | 0.462 |
|  | Calcium, mmol/L | 2.14 (2.02, 2.31) | 2.11 (2.01, 2.23) | 0.666 |
| Stroke evaluation | |  |  |  |
|  | NIHSS at baseline | 14 (11, 19) | 14 (10, 18) | 0.569 |
|  | ASPECT at baseline | 8 (7, 9) | 8 (7, 9) | 0.806 |
| Site of vessel occlusion，n(%) | | |  |  |
|  | ICA | 95 (39.4%) | 74 (38.5%) | 0.739 |
|  | M1-MCA | 124 (51.5%) | 104 (54.2%) |  |
|  | Others | 22 (9.1%) | 14 (7.3%) |  |
| Procedure | | |  |  |
|  | Onset to puncture time, min | 340 (240, 415) | 335 (255, 410) | 0.662 |
|  | Onset to recanalization, min | 380 (320, 470) | 385 (320, 450) | 0.528 |
|  | Successful recanalization, n (%) | 207 (85.9%) | 172 (89.6%) | 0.248 |
|  | Intravenous thrombolysis, n (%) | 139 (57.7%) | 123 (64.1%) | 0.177 |
|  | Number of passes | 2 (1, 2) | 2 (1, 2) | 0.763 |
|  | sICH, n(%) | 35 (14.5%) | 23 (12.0%) | 0.440 |
| TOAST subtype, n (%) | |  |  |  |
|  | Large-artery atherosclerosis | 104 (43.2%) | 86 (44.8%) | 0.533 |
|  | Cardioembolism | 105 (43.6%) | 87 (45.3%) |  |
|  | Other determined/undetermined | 32 (13.3%) | 19 (9.9%) |  |

Abbreviation, ICA, internal carotid artery, M1-MCA, M1 of middle cerebral artery, NIHSS, National Institutes of Health Stroke Scale, ASPECT, Alberta Stroke Program Early CT Score, sICH, symptomatic intracranial hemorrhage, TOAST, Trial of Org 10172 in Acute Stroke Treatment.

**Supplementary Table 2.** **Baseline characteristics of the patients according to quartiles of albumin corrected calcium level**

| Variables | | Albumin-corrected calcium level, mmol | | | | *P* |
| --- | --- | --- | --- | --- | --- | --- |
|  |  | Q1 (<2.08) | Q2 (2.08-2.18) | Q3 (2.18-2.29) | Q4 (>2.29) |  |
| Age, years | | 74.0 (63.5, 77.0) | 74.0 (64.0, 79.0) | 71.0 (65.0, 79.0) | 73.0 (64.0, 81.0) | 0.438 |
| Male, n (%) | | 32 (66.7%) | 33 (68.8%) | 32 (66.7%) | 35 (72.9%) | 0.901 |
| Risk factors, n (%) | |  |  |  |  |  |
|  | Hypertension | 29 (60.4%) | 37 (77.1%) | 29 (60.4%) | 33 (68.8%) | 0.248 |
|  | Diabetes mellitus | 16 (33.3%) | 14 (29.2%) | 14 (29.2%) | 13 (27.1%) | 0.925 |
|  | Dyslipidemia | 32 (66.7%) | 31 (64.6%) | 30 (62.5%) | 28 (58.3%) | 0.854 |
|  | Ischemic cardiopathy | 12 (25.0%) | 17 (35.4%) | 14 (29.2%) | 15 (31.3%) | 0.733 |
|  | Arrhythmia | 16 (33.3%) | 18 (37.5%) | 22 (45.8%) | 22 (45.8%) | 0.506 |
|  | Stroke history | 9 (18.8%) | 10 (20.8%) | 11 (22.9%) | 11 (22.9%) | 0.952 |
|  | Hypertension med use | 12 (25.0%) | 28 (58.3%) | 18 (37.5%) | 16 (33.3%) | **0.007** |
|  | Diabetes med use | 7 (14.6%) | 8 (16.7%) | 7 (14.6%) | 3 (6.3%) | 0.438 |
|  | Smoking | 21 (43.8%) | 16 (33.3%) | 19 (39.6%) | 24 (50.0%) | 0.405 |
|  | Drinking | 9 (18.8%) | 9 (18.8%) | 5 (10.4%) | 9 (18.8%) | 0.615 |
| Laboratory results | |  |  |  |  |  |
|  | Fasting glucose, mmol/L | 7.5 (5.9, 9.4) | 6.7 (5.8, 8.1) | 6.7 (5.4, 10.1) | 6.5 (5.2, 9.3) | 0.494 |
|  | Triglycerides, mmol/L | 1.27 (0.87, 1.62) | 1.37 (0.99, 1.80) | 1.37 (0.95, 1.80) | 1.44 (0.83, 1.79) | 0.794 |
|  | Total cholesterol, mmol/L | 4.36 (3.68, 5.22) | 4.20 (3.59, 5.04) | 4.26 (3.61, 5.39) | 4.15 (3.50, 5.16) | 0.917 |
|  | Homocysteine, mmol/L | 11.5 (9.2, 14.4) | 16.0 (10.5, 21.2) | 12.4 (10.0, 18.2) | 14.5 (10.8, 17.6) | **0.011** |
|  | C-reactive protein, mg/L | 5.9 (4.1, 18.0) | 8.3 (3.0, 19.2) | 13.7 (3.4, 36.7) | 7.7 (3.7, 19.9) | 0.194 |
|  | Albumin, g/L | 37.5 (35.6, 44.5) | 37.2 (35.6, 39.8) | 37.6 (34.1, 40.3) | 38.4 (33.1, 40.4) | 0.893 |
| Stroke evaluation | |  |  |  |  |  |
|  | ICA occlusion，n(%) | 19 (39.6%) | 16 (33.3%) | 12 (25.0%) | 27 (56.3%) | **0.014** |
|  | NIHSS at baseline | 13 (8, 15) | 12 (10, 18) | 14 (10, 19) | 14 (10, 18) | 0.228 |
|  | ASPECT at baseline | 8 (7, 9) | 8 (7, 9) | 8 (7, 10) | 8 (7, 8) | 0.379 |

ICA, internal carotid artery, NIHSS, National Institutes of Health Stroke Scale, ASPECT, Alberta Stroke Program Early CT Score, sICH, symptomatic intracranial hemorrhage.

**Supplementary Table 3. Characteristics of patients with or without arterial revascularization**

| Variables | | Revascularization (n=172) | No revascularization  (n=20) | *P* |
| --- | --- | --- | --- | --- |
| Age, years | | 71.9 ± 10.7 | 69.2 ± 10.7 | 0.279 |
| Male, n (%) | | 118 (68.6%) | 17 (70.0%) | 0.899 |
| Risk factors, n (%) | |  |  |  |
|  | Hypertension | 116 (67.4%) | 12 (60.0%) | 0.504 |
|  | Diabetes mellitus | 46 (26.7%) | 11 (5.0%) | 0.009 |
|  | Dyslipidemia | 111 (64.5%) | 10 (50.0%) | 0.203 |
|  | Ischemic cardiopathy | 54 (31.4%) | 4 (20.0%) | 0.293 |
|  | Arrhythmia | 74 (43.0%) | 4 (20.0%) | 0.047 |
|  | Stroke history | 32 (18.6%) | 9 (45.0%) | 0.006 |
|  | Hypertension med use | 66 (38.4%) | 8 (40.0%) | 0.887 |
|  | Diabetes med use | 21 (12.2%) | 4 (20.0%) | 0.327 |
|  | Smoking | 67 (39.0%) | 13 (65.0%) | 0.025 |
|  | Drinking | 27 (15.7%) | 5 (25.0%) | 0.222 |
| Laboratory results | |  |  |  |
|  | Fasting glucose, mmol/L | 6.6 (5.5, 8.7) | 6.5 (5.6, 9.5) | 0.947 |
|  | Triglycerides, mmol/L | 1.30 (0.93, 1.79) | 1.49 (1.01, 1.92) | 0.138 |
|  | Total cholesterol, mmol/L | 4.31 (3.64, 5.17) | 4.10 (3.30, 4.51) | 0.329 |
|  | Homocysteine, mmol/L | 13.2 (10.5, 17.6) | 10.9 (9.3, 16.0) | 0.038 |
|  | C-reactive protein, mg/L | 7.9 (3.7, 18.6) | 16.0 (3.8, 44.5) | 0.150 |
|  | Albumin, g/L | 37.6 (35.0, 40.4) | 37.9 (35.0, 40.8) | 0.895 |
|  | Calcium, mmol/L | 2.11 (2.02, 2.23) | 2.28 (2.04, 2.42) | 0.020 |
| Stroke evaluation | |  |  |  |
|  | ICA occlusion，n(%) | 61 (35.5%) | 13 (65.0%) | 0.010 |
|  | NIHSS at baseline | 14 (10, 18) | 14 (10, 17) | 0.626 |
|  | ASPECT at baseline | 8 (7, 9) | 8 (6, 9) | 0.538 |
|  | Intravenous thrombolysis, n (%) | 112 (65.1%) | 11 (55.0%) | 0.372 |
|  | Number of passes | 1 (1, 2) | 2 (1.5, 3.5) | 0.035 |
| TOAST subtype, n (%) | |  |  |  |
|  | Large-artery atherosclerosis | 72 (41.9%) | 14 (70.0%) | 0.070 |
|  | Cardioembolism | 83 (48.3%) | 4 (20.0%) |  |
|  | Other determined/undetermined | 17 (9.9%) | 2 (10.0%) |  |

ICA, internal carotid artery, NIHSS, National Institutes of Health Stroke Scale, ASPECT, Alberta Stroke Program Early CT Score, sICH, symptomatic intracranial hemorrhage, TOAST, Trial of Org 10172 in Acute Stroke Treatment.

**Supplementary Table 4. Characteristics of patients with good or poor outcome**

| Variables | | Good outcome  (n=66) | Poor outcome  (n=110) | *P* |
| --- | --- | --- | --- | --- |
| Age, years | | 70.1 ± 10.8 | 72.8 ± 10.1 | 0.096 |
| Male, n (%) | | 48 (72.7%) | 74 (67.3%) | 0.447 |
| Risk factors, n (%) | |  |  |  |
|  | Hypertension | 38 (57.6%) | 78 (70.9%) | 0.071 |
|  | Diabetes mellitus | 14 (21.2%) | 37 (33.6%) | 0.079 |
|  | Dyslipidemia | 39 (59.1%) | 73 (66.4%) | 0.332 |
|  | Ischemic cardiopathy | 11 (16.7%) | 41 (37.3%) | 0.004 |
|  | Arrhythmia | 20 (30.8%) | 52 (47.3%) | 0.027 |
|  | Stroke history | 10 (15.2%) | 29 (26.4%) | 0.083 |
|  | Hypertension med use | 30 (30.3%) | 46 (41.8%) | 0.127 |
|  | Diabetes med use | 5 (7.6%) | 19 (17.3%) | 0.070 |
|  | Smoking | 18 (27.3%) | 53 (48.2%) | 0.006 |
|  | Drinking | 7 (10.6%) | 22 (20.0%) | 0.104 |
| Laboratory results | |  |  |  |
|  | Fasting glucose, mmol/L | 7.1 (5.5, 8.9) | 6.5 (5.4, 8.9) | 0.632 |
|  | Triglycerides, mmol/L | 1.29 (0.97, 1.59) | 1.43 (0.89, 1.92) | 0.450 |
|  | Total cholesterol, mmol/L | 4.30 (3.44, 5.32) | 4.25 (3.72, 5.06) | 0.504 |
|  | Homocysteine, mmol/L | 11.4 (9.6, 17.1) | 13.0 (10.9, 17.9) | 0.038 |
|  | C-reactive protein, mg/L | 6.4 (3.1, 23.9) | 7.7 (3.7, 19.8) | 0.848 |
|  | Albumin, g/L | 37.4 (35.1, 39.7) | 37.7 (35.3, 41.3) | 0.297 |
|  | Calcium, mmol/L | 2.06 (1.98, 2.21) | 2.17 (2.06, 2.31) | 0.001 |
| Stroke evaluation | |  |  |  |
|  | ICA occlusion，n(%) | 17 (25.8%) | 52 (47.3%) | 0.005 |
|  | NIHSS at baseline | 11 (8, 15) | 15 (13, 18) | < 0.001 |
|  | ASPECT at baseline | 8 (7, 10) | 8 (7, 9) | 0.024 |
|  | Successful recanalization, n (%) | 62 (93.9%) | 96 (87.3%) | 0.158 |
|  | Onset to recanalization, min | 380 (318, 455) | 380 (310, 490) | 0.205 |
|  | Intravenous thrombolysis, n (%) | 49 (74.2%) | 64 (58.2%) | 0.031 |
|  | Number of passes | 1 (1, 2) | 2 (1, 3) | < 0.001 |
|  | sICH, n(%) | 1 (1.5%) | 19 (17.3%) | 0.001 |
| TOAST subtype, n (%) | |  |  |  |
|  | Large-artery atherosclerosis | 34 (51.5%) | 45 (40.9%) | 0.344 |
|  | Cardioembolism | 27 (40.9%) | 52 (47.3%) |  |
|  | Other determined/undetermined | 5 (7.6%) | 13 (11.8%) |  |

ICA, internal carotid artery, NIHSS, National Institutes of Health Stroke Scale, ASPECT, Alberta Stroke Program Early CT Score, sICH, symptomatic intracranial hemorrhage, TOAST, Trial of Org 10172 in Acute Stroke Treatment.
